# Scale-Dependent Effects of a Heterogeneous Landscape on Genetic Differentiation in the Central American Squirrel Monkey (*Saimiri oerstedii*)

**DOI:** 10.1371/journal.pone.0043027

**Published:** 2012-08-15

**Authors:** Mary E. Blair, Don J. Melnick

**Affiliations:** 1 Department of Ecology, Evolution and Environmental Biology, Columbia University, New York, New York, United States of America; 2 New York Consortium in Evolutionary Primatology, New York, New York, United States of America; Instituto de Higiene e Medicina Tropical, Portugal

## Abstract

Landscape genetic studies offer a fine-scale understanding of how habitat heterogeneity influences population genetic structure. We examined population genetic structure and conducted a landscape genetic analysis for the endangered Central American Squirrel Monkey (*Saimiri oerstedii*) that lives in the fragmented, human-modified habitats of the Central Pacific region of Costa Rica. We analyzed non-invasively collected fecal samples from 244 individuals from 14 groups for 16 microsatellite markers. We found two geographically separate genetic clusters in the Central Pacific region with evidence of recent gene flow among them. We also found significant differentiation among groups of *S. o. citrinellus* using pairwise F_ST_ comparisons. These groups are in fragments of secondary forest separated by unsuitable “matrix” habitats such as cattle pasture, commercial African oil palm plantations, and human residential areas. We used an individual-based landscape genetic approach to measure spatial patterns of genetic variance while taking into account landscape heterogeneity. We found that large, commercial oil palm plantations represent moderate barriers to gene flow between populations, but cattle pastures, rivers, and residential areas do not. However, the influence of oil palm plantations on genetic variance was diminished when we restricted analyses to within population pairs, suggesting that their effect is scale-dependent and manifests during longer dispersal events among populations. We show that when landscape genetic methods are applied rigorously and at the right scale, they are sensitive enough to track population processes even in species with long, overlapping generations such as primates. Thus landscape genetic approaches are extremely valuable for the conservation management of a diverse array of endangered species in heterogeneous, human-modified habitats. Our results also stress the importance of explicitly considering the heterogeneity of matrix habitats in landscape genetic studies, instead of assuming that all matrix habitats have a uniform effect on population genetic processes.

## Introduction

Many species exist in spatially structured populations linked by dispersal and gene flow, which can influence evolutionary, demographic, and ecological processes. Studies of population genetic structure are important for species living in complex, heterogeneous landscapes, which may affect that structure [1]. Understanding population genetic structure is also critical to informing conservation management [2]; conservation managers need to measure the extent and distribution of genetic diversity to accurately predict population persistence, especially for small, fragmented populations [3].

Landscape genetic approaches are increasingly used to understand the influence of landscape characteristics on population genetic structure and dispersal patterns [4], [5], [6], [7]. These emerging approaches combine population genetics, spatial statistics, and landscape ecology to measure the effects of landscape features on gene flow [8], [9], [10]. Landscape genetic approaches attempt to detect genetic discontinuities among individuals and then correlate those discontinuities with landscape features [9]. For example, many recent landscape genetic studies of birds, herpetofauna, terrestrial mammals, and primates found that geographic distances incorporating a cost to particular landscape features showed a stronger correlation to genetic distances than straight-line Euclidean distances between sampled individuals [11], [12], [13], [14], [15], [16], [17].

One particular challenge in landscape genetics has been to quantify the relative effects of various landscape parameters on gene flow [4], [18], [19]. Recent studies in landscape ecology reject simplistic models where the matrix, or the unsuitable habitat between patches of suitable habitat for a given species, uniformly inhibits movement among patches. Instead, the matrix is dynamic, heterogeneous, and can have both positive and negative effects on dispersal and thus the long-term persistence of a species [20], [21], [22], [23], [24], [25], [26], [27]. Responses to matrix quality and heterogeneity are species-specific, often correlating with body size, degree of arboreality, dietary specialization, and habitat breadth [28], [29], [30]. Thus, landscape genetic studies should compare different classes of matrix habitats at multiple scales to understand their relative effects on genetic variation and better predict processes of population divergence in modified landscapes.

The endangered Central American Squirrel Monkey (*Saimiri oerstedii*, Primates: Cebidae) provides an ideal opportunity to investigate population genetic structure in a human modified, heterogeneous landscape. *S. oerstedii* live in groups of 18 or more individuals, which have home ranges of approximately 200 ha. Their diet includes arthropods, flowers, fruits, and small vertebrates [31], [32], and they are restricted to the Pacific moist forests of Costa Rica and northern Panama below ∼500 m asl [31], [33], [34], [35]. This range area is characterized by frequent landscape disturbance from high rainfall, wind, hurricanes, and rugged topography [36], [37]. The subspecies *S. o. citrinellus* inhabits a particularly heterogeneous landscape in the Central Pacific region of Costa Rica, where fruit and rice plantations and cattle pasture replaced approximately 80% of the natural forest in the early 1900s [38].

Despite this heterogeneity, little work has been done to determine the effects of such drastic natural and anthropogenic landscape change on the genetic structure of *S. o. citrinellus* populations. To date the only published study of genetic diversity in *S. o. citrinellus* was based on a small sample (N = 8) from Manuel Antonio National Park (MANP) [39], which is the smallest national park in Costa Rica and the only protected area within the range of *S. o. citrinellus*. The study concluded that *S. oerstedii* have moderate to high genetic diversity compared to the three other primate species in Costa Rica (*Alouatta palliata, Ateles geoffroyi,* and *Cebus capucinus*). A much larger sample collected across a broader spatial scale and wider range of habitats is necessary to determine the effects of landscape heterogeneity on *S. o. citrinellus* population genetic structure. In this study, we analyzed a large number of non-invasively collected molecular samples of *S. o. citrinellus* and characterized overall population genetic structure using Bayesian clustering algorithms and F-statistics. We also used fine-scale landscape data within a least-cost distance framework to determine whether there is a relationship between landscape heterogeneity and population genetic structure and which, if any, matrix habitats might be related to genetic structure in *S. o. citrinellus.* Several aspects of *S. oerstedii* behavioral ecology suggest that some types of matrix habitat will affect patterns of gene flow more than others. For example, *S. oerstedii* are known to traverse small fruit plantations and live fences around residential areas, while they likely do not traverse large commercial oil palm plantations and rice plantations [32], [35]. If some matrix habitats represent barriers to gene flow while others do not, geographic distances that weight barrier matrix habitats with high costs should correlate more strongly with genetic distance than geographic distances that weight passable matrix habitats with high costs. By contrast, if all matrix habitats prevent gene flow, different least-cost measures of distance through matrix habitat should not differ in the strength of their associations with genetic distance.

## Methods

### Ethics Statement

Permits to collect and import *S. o. citrinellus* fecal samples included Costa Rican Ministry of Energy and the Environment permit ACOPAC-INVN-14-08 and Centers for Disease Control and Prevention permit 2008-08-151. Also, an IACUC animal care protocol was approved by Columbia University for this research (AC-AAAA5583).

### Sampling and DNA Extraction

**Figure 1:**
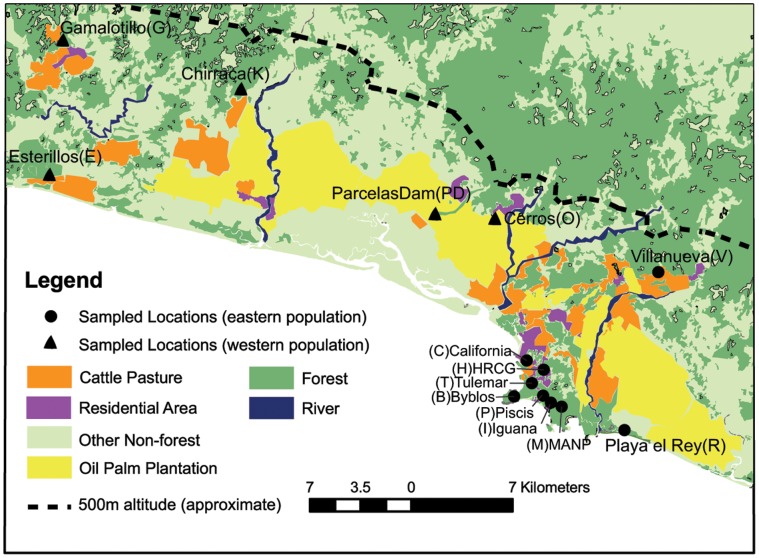
Sampled *S. o. citrinellus* groups in the Central Pacific region of Costa Rica. Sampled *S. o. citrinellus* groups in the Central Pacific region of Costa Rica, showing the limit of 500 m asl to their distribution and different classes of matrix habitat as defined by a manual land cover classification.

Fecal samples were collected from *S. o. citrinellus* in the Central Pacific region of Costa Rica from September 2008 – April 2009 (Figure 1). *S. o. citrinellus* is not continuously distributed between sampling locations as they are restricted to larger secondary forest fragments [33], [40]; two groups (PD and O) were sampled in oil palm plantations but adjacent to a forest fragment or riparian forest. From 304 fecal samples, we verified genotypes for 233 *S. o. citrinellus* individuals, comprised of 10 to 20 adult individuals from each of 14 groups. The average size of sampled groups was 39 individuals (range 18–67). Whenever possible (N = 13 groups), more than 10 individuals per group were sampled to increase the precision of genetic analyses in detecting dispersal and migration [41]. Samples were stored in 8 ml plastic tubes with *RNAlater* buffer (Ambion) at −4°C in the field and −20°C in the laboratory. Eleven additional DNA samples from individuals of the southern subspecies *S. o. oerstedii*, used as an outgroup, were contributed by G. Gutierrez (University of Costa Rica) for a total of 244 samples.

We extracted DNA using QIAamp DNA Stool Minikits (Qiagen) with small modifications to the “Isolation of DNA from Stool for Human DNA Analysis” protocol (see [42], [43]). We used real-time quantitative PCR after extraction to quantify the amount of nuclear DNA in each sample with iQ SYBR Green Supermix (Bio-Rad) and universal primate primers amplifying approximately 200 bp of nuclear DNA [44]. We included samples with greater than 0.5 ng/µl DNA concentrations (averaged over two replicate runs) in our genotyping analyses.

### Microsatellite Genotyping

We PCR amplified 17 autosomal microsatellite markers on multiplex panels of three or four markers (CJ7 [45]; D17s804, D3s1210, D3s1229, D3s1776, D4s111, D5s111, D8s165, D8s260 [46]; Leon 15, Leon 21 [47]; LL118, LL157, LL311 [48]; Locus5 [49]; SB38 [50]; Table S1) using Multiplex PCR Kits (Qiagen; for reaction concentrations and conditions see [43]). PCR products were electrophoresed on an ABI 3730 DNA Analysis System with GENESCAN 500 ROX size standard, and genotypes were called using GeneMapper software (ABI). We confirmed heterozygous genotypes by scoring alleles at least four times and homozygous genotypes at least seven times since allelic dropout is often a problem when amplifying microsatellite markers from fecal samples [51], [52], [53]. We used the program MICROCHECKER [54] to test for null alleles, and removed one of the 17 microsatellite markers because it was found to possibly contain null alleles (D13s160). We tested for linkage disequilibrium and deviations from Hardy-Weinberg equilibrium (HWE) across markers in ARLEQUIN v 3.1 [55] and found no evidence for linkage disequilibrium. We did find violations of HWE when analyzing samples at the species and population levels, consistent with a Wahlund effect [56], [57]. At the group level, three groups had one marker that was significantly out of HWE (D3s1766 at Gamalotillo, Leon21 at Chirraca, and Leon15 at MANP), likely due to the presence of related individuals in the sample [58], [59], [60].

### Analysis of Population Genetic Structure

We ran our multilocus genotypes in STRUCTURE v 2.2 [61] and BAPS v 2 [62] to infer the number of genetic clusters in our dataset. We ran 10 independent iterations of K = 1−16 in STRUCTURE for 2,000,000 Markov Chain Monte Carlo (MCMC) generations with a 200,000 burn-in period, assuming correlated allele frequencies and admixture. We inferred K using ln *P*(X | K) and the ΔK method [63], where optimum K has the highest ΔK value, or rate of change in the log probability of the data between successive K-values. We ran BAPS under the individual clustering module and the default settings (stochastic optimization) also for 10 separate iterations for a maximum K of 1–21. Both programs were run without spatial information.

We also examined genetic structure in *S. o. citrinellus* microsatellite data with pairwise tests for differentiation among groups and populations using F-statistics [64] calculated with Weir and Cockerham’s [65] estimators in FSTAT v 2.9 [66], for 10,000 randomizations not assuming HWE. Bonferroni corrections were used throughout when conducting multiple comparisons.

We detected first generation migrants and admixed individuals using STRUCTURE and GENECLASS v 2.0 [67], [68]. Migrants were defined as individuals that were assigned to one population but geographically sampled from another population. Admixed individuals were defined as those individuals that could not be confidently assigned to either population following the ranking and plotting approach of Beaumont *et al.* [69]. To detect first generation migrants in STRUCTURE, we ran the program using the cluster memberships inferred as described above using the ΔK method as prior population information. We conducted several runs using a range of values for MIGRPRIOR (0.001−0.1) following Pritchard *et al.* [70]. Because choice of MIGRPRIOR did not significantly affect program outputs, we present results from MIGRPRIOR  =  0.09, the average migration rate between populations of *S. o. citrinellus* found using the software BAYESASS [71], following Liu *et al.* [16]. Burn-in and run length were the same as earlier runs of STRUCTURE without prior population information. We also performed an exclusion test and used the ‘Detect first generation migrants’ option in GENECLASS [68], [72], using both L_h_ and L_h_/L_max_, which represent, respectively, the most appropriate statistic when all potential source populations have not been sampled and when they have [72]. The probability of individual genotypes coming from each population was calculated by comparing individual genotypes to 10,000 simulated individuals per population [72].

We tested for genetic signatures of a recent population bottleneck using BOTTLENECK [73]. We tested our data under the Infinite Alleles Model (IAM), the Stepwise Mutation Model (SSM), and the Two Phase Model (TPM) with 10,000 replications using a sign test. The sign test compares observed and expected heterozygosity excess. If excess is higher than expected (based on equilibrium) for a large majority of markers in a population, the population may have recently experienced a genetic bottleneck [73], [74].

### Land Cover Classification

Using 32 geo-referenced aerial photographs (taken with a DCS camera, each 10×10.5 km), a 90×7 km multispectral MASTER line image taken in the year 2005 obtained from the Centro Nacional de Alta Technología (CENAT) in Costa Rica, and a forest cover dataset generated by EOSL, CCT and FONAFIFO [75] with Landsat 7 TM satellite imagery from the year 2000, we delineated five habitat classes (forest, cattle pasture, rivers, oil palm plantations, and residential areas; Figure 1) manually in ArcGIS v 9.3 (ESRI) at a 20×20 m resolution for the 1800 km^2^ study area. We defined residential areas as clusters of human residences less than 100 m apart and consisting of a total area greater than 3 hectares. Our classification also included an “other non-forest” category, which included shrimp farms, rice plantations, abandoned lots, and residences that were not concentrated enough to fit our definition of a residential area. Ninety-eight percent of 131 ground reference points were accurately reflected in the classification.

**Figure 2:**
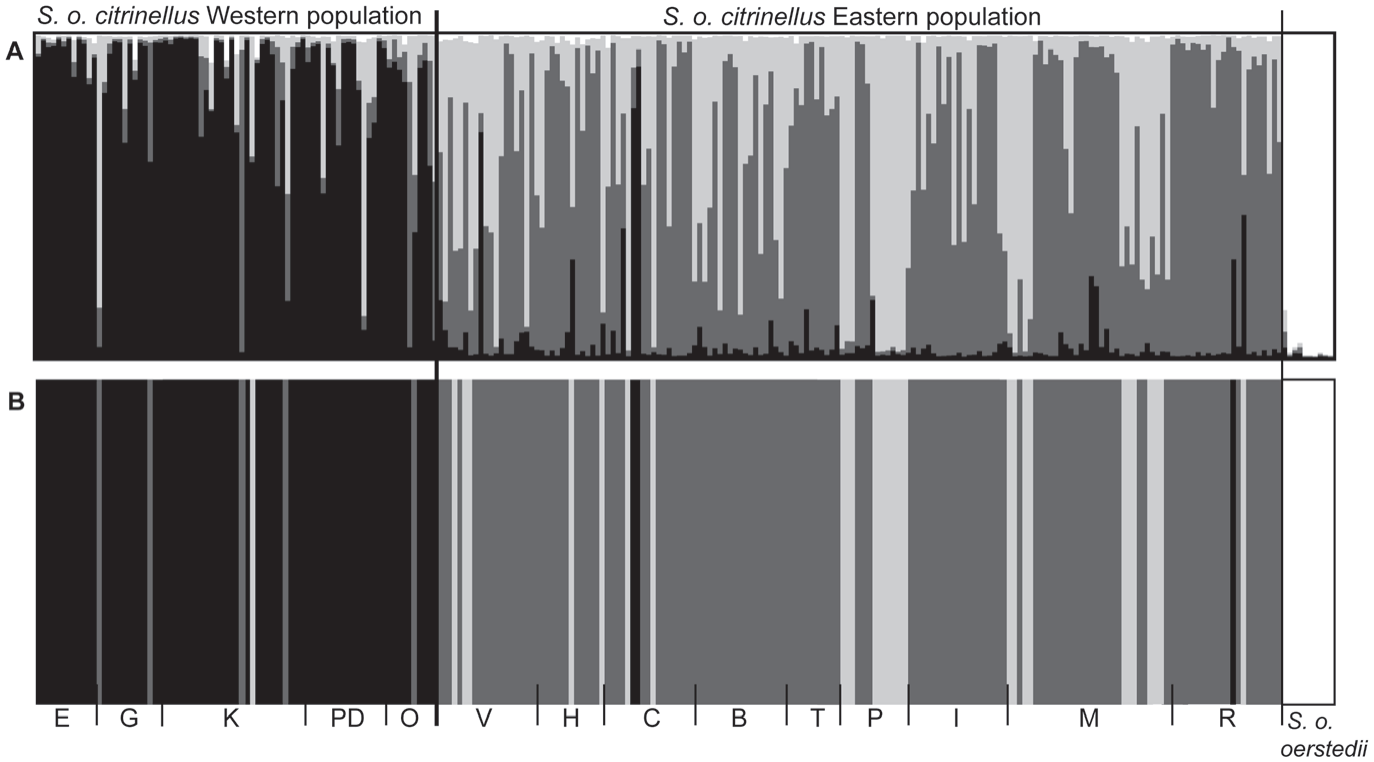
Distribution of three genetic clusters estimated in STRUCTURE (A) and BAPS (B). Distribution of three genetic clusters estimated in STRUCTURE (A) and BAPS (B). Vertical lines are broken into colored segments showing the proportion of each individual assigned to each K (within *S. o. citrinellus*: western cluster – black, eastern cluster 1– light grey, eastern cluster 2– grey; *S. o. oerstedii* – white). Sample locations are listed at the bottom of the figure, and are arrayed in west to east order (left to right).

### Landscape Genetic Analyses

We estimated pairwise genetic relationships between individuals using Rousset’s *â* [76] and Moran’s *I* [77], [78] to measure both distance (*â*) and similarity (*I*) [30]. We measured two types of geographic distances: Euclidean distances, straight-line distances on a map, and least-cost geographic distances, where the costs of dispersing across different habitat classes were incorporated into the measure of geographic distance. We calculated Euclidean geographic distances in ArcMap v 9.3 (ESRI) using the sampled coordinates and we calculated least-cost geographic distances for the five habitat classes identified above (forests, oil palm plantations, cattle pastures, residential areas, and rivers) using the COSTDISTANCE function in ArcGIS v 9.3 (ESRI). We varied the cost of one class while keeping all others at an equal, low cost (1) and then repeated this process for each habitat class. We assessed least-cost distances for a range of 6 arbitrary cost values (10, 50, 100, 1000, 5000, 10000) to account for sensitivity [79], [80].

We performed Mantel tests of matrix correspondence [81] between genetic distances and geographic distances in ZT [82]. Also, because least-cost distances and Euclidean distances are not independent, we performed partial Mantel tests [83] in ZT to test the strength of relationships between genetic and least-cost distance matrices while controlling for the effect of Euclidean distance [84], [85]. Partial correlations show high power and accuracy in their ability to infer the effect of landscapes on dispersal when there is a strong contrast between the permeability of different landscape elements [86]. Significance was assessed with 10,000 permutations.

Recent studies have suggested that the spatial scale of landscape genetic analyses can have important effects on results, especially inferences about which landscape features affect gene flow [87], [88], [89]. To test the stability of our inferences across spatial scales, we repeated the above analyses including only pairs of samples within genetic clusters of *S. o. citrinellus* as inferred by analyses of population genetic structure, excluding between population pairs.

We used the results of our least-cost distance analyses to generate a resistance surface characterizing the cumulative effects of landscape heterogeneity on gene flow in *S. o. citrinellus*, implemented in the software CIRCUITSCAPE 3.5 [90]. Instead of calculating a single least-cost path, CIRCUITSCAPE incorporates aspects of electronic circuit theory (i.e. electronic resistance) with a random walk approach to visualize resistance patterns across the landscape [91], [92], [93]. We tested for relationships between pairwise resistance distances (generated using the “pairwise” mode in CIRCUITSCAPE) and genetic distances using simple and partial Mantel tests as described above. Also, we produced cumulative current flow maps in the “all to one” mode, with focal points as sampled groups and an 8-neighbor connection scheme, and the source current for each group scaled to group size. The habitat grid encompassed a 5–25 km buffer around peripheral focal points, with forests, rivers, residential areas and cattle pastures at very low costs (<10) and oil palm plantations at a moderate cost of 20, following recommendations from the CIRCUITSCAPE manual (resistance values above 20 are considered moderate, while values above 200 are considered high). We ran the program under several other parameterizations with similar results to those presented.

## Results

### Analysis of Population Genetic Structure

Bayesian clustering analysis revealed at least two genetically distinct populations within *S. o. citrinellus* in the Central Pacific of Costa Rica. The most likely number of clusters across the whole sample, which included outgroup *S. o. oerstedii* individuals, was four in both STRUCTURE and BAPS (Figure 2,S1), although K = 2 or 3 were only slightly less likely (Figure S1). For K = 4, one cluster represents samples from the subspecies *S. o. oerstedii* while the other three clusters are within *S. o. citrinellus*. The first cluster within *S. o. citrinellus* includes almost all individuals from western groups. The other two clusters do not seem to be geographically separated and include mostly members from eastern groups (Figure 2). The two eastern clusters likely represent ancestral polymorphism that has not been sorted out in this population. We defined a western population and an eastern population of *S. o. citrinellus* based on the strong geographic clustering inferred using STRUCTURE and BAPS. We also found significant population differentiation using F-statistics between the defined eastern and western populations of *S. o. citrinellus* (F_ST_ = 0.0903, *P* = 0.05). Microsatellite allelic diversity in populations of *S. o. citrinellus* ranged from 3 to 15 alleles (mean = 8.1) in the western population and from 5 to 27 (mean = 10.6) in the eastern population. Pairwise F_ST_ values among groups of *S. o. citrinellus* ranged from 0.016–0.19, with a mean of 0.103 (Table 1). Pairwise F_ST_ values among groups from the same population (mean = 0.06, range 0.016–0.11) were less than pairwise F_ST_ values among groups from different populations (mean = 0.14, range 0.070–0.19, Table 1).

**Table 1:** Pairwise F_ST_ values among groups of *S. o. citrinellus*.

	Western Groups					Eastern Groups								
	E	G	K	O	PD	B	C	H	I	M	P	R	T	V
E	–	0.0502	0.0436	0.0783	0.0971	0.1621	0.1374	0.1375	0.145	0.1529	0.1781	0.1834	0.1679	0.1247
G	*	–	0.0656	0.0992	0.1128	0.1299	0.1375	0.1293	0.1233	0.1183	0.1569	0.1515	0.1433	0.1294
K	NS	*	–	0.0705	0.1095	0.1544	0.138	0.1513	0.1505	0.1353	0.173	0.1747	0.1595	0.1398
O	*	*	NS	–	0.0584	0.1222	0.0756	0.1152	0.1013	0.1032	0.1535	0.1436	0.1041	0.07
PD	*	*	*	*	–	0.1345	0.1207	0.1262	0.1171	0.1385	0.1919	0.1909	0.1368	0.0997
B	*	*	*	*	*	–	0.0502	0.0529	0.0438	0.0533	0.0972	0.0659	0.0453	0.0475
C	*	*	*	NS	*	*	–	0.0629	0.0351	0.0246	0.0647	0.0643	0.0405	0.0364
H	*	*	*	*	*	*	*	–	0.0193	0.061	0.1088	0.0714	0.0428	0.0312
I	*	*	*	*	*	*	*	NS	–	0.0306	0.0888	0.056	0.0163	0.0299
M	*	*	*	*	*	*	NS	*	*	–	0.0638	0.0671	0.0171	0.0517
P	*	*	*	*	*	*	*	*	*	NS	–	0.1123	0.1002	0.0911
R	*	*	*	*	*	*	*	*	*	*	*	–	0.078	0.0844
T	*	*	*	*	*	NS	*	*	NS	NS	*	*	–	0.0424
V	*	*	*	*	*	*	*	*	*	*	*	*	*	–
*N*	12	13	28	10	14	18	18	13	20	22	13	21	11	20

The last row shows sample sizes (*N)* for each group.

NS  =  non significant *P* value.

* = *P*<0.05 (under a Bonferroni corrected threshold of 0.0005).

Analyses using STRUCTURE and GENECLASS estimated 7 likely migrants and 10 potentially admixed individuals between the western and eastern populations (Table 2). STRUCTURE estimated 1 potential migrant (individual G1, *P* = 0.019), while GENECLASS estimated the same individual as a migrant in addition to 6 others (*P*<0.01) using both likelihood methods (L_h_ and L_h_/L_max_). These 7 migrants were assigned to a cluster where they were not geographically sampled in GENECLASS and also had lower probabilities of belonging to their geographic origin cluster compared to other individuals in STRUCTURE (Table 2). Ten to 40 km separated sampled sites and inferred populations of origin.

**Table 2:** Results of migrant detection analyses.

Sample	Geographic origin	STRUCTURE *Q* (N/S clusters)	GENECLASS cluster of highest probability assignment-exclusion test	GENECLASS highest assignment probability	GENECLASS F_0_ migrant probability (L_h_;L_h_/L_max_ indicated with ∧, *P<0.01)	STRUCTURE probability belongs to origin cluster	Final migrant (M)/admixture (AD) classification
G1	Western	0.034/0.964	Eastern	0.0575	0.9943∧*	0.019	M
O16	Western	0.033/0.963	Eastern	0.8108	0.9994∧*	0.061	M
PD7	Western	0.086/0.891	Eastern	0.0491	0.9949∧*	0.743	M
O6	Western	0.595/0.402	Eastern	0.7748	0.9947∧*	0.828	M
O2	Western	0.388/0.608	Western/Eastern	0.1092/0.2380	0.9866	0.506	AD
K1	Western	0.018/0.979	Western/Eastern	0.0532/0.0817	NS	0.046	AD
PD8	Western	0.680/0.307	Western/Eastern	0.8376/0.7533	NS	0.877	AD
K11	Western	0.606/0.388	Western/Eastern	0.0968/0.1132	NS	0.879	–
K4	Western	0.176/0.814	Western/Eastern	0.1794/0.2430	NS	0.945	AD
K17	Western	0.531/0.467	Western/Eastern	0.0426/0.0234	NS	0.948	–
PD2	Western	0.658/0.339	Western/Eastern	0.2370/0.2449	NS	0.951	AD
K20	Western	0.683/0.313	Western	0.0812	NS	0.967	–
G14	Western	0.665/0.328	Western/Eastern	0.0410/0.0587	NS	0.977	–
PD13	Western	0.509/0.485	Western/Eastern	0.0389/0.0146	NS	0.992	–
G6	Western	0.608/0.387	Western	0.0217	NS	0.993	–
PD9	Western	0.728/0.263	Western/Eastern	0.2002/0.1078	NS	0.993	–
K19	Western	0.697/0.242	Western	0.0131	NS	0.999	–
C21	Eastern	0.903/0.094	Western	0.6139	0.9946∧*	0.242	M
C19	Eastern	0.400/0.589	Western	0.2295	0.9976∧*	0.508	M
V17	Eastern	0.698/0.299	Western	0.0162	0.9985∧*	0.62	M
C20	Eastern	0.772/0.223	Western/Eastern	0.5680/0.6632	0.9795	0.474	AD
R1	Eastern	0.442/0.552	Western/Eastern	0.0080/0.1104	0.9864	0.831	AD
H26	Eastern	0.305/0.643	Western/Eastern	0.0001/0.0001	0.9874	0.95	–
R2	Eastern	0.305/0.692	Western/Eastern	0.3095/0.4535	NS	0.547	AD
M5	Eastern	0.221/0.772	Western/Eastern	0.1232/0.3765	NS	0.766	AD
M6	Eastern	0.253/0.743	Western/Eastern	0.2001/0.5669	NS	0.849	AD

In STRUCTURE, we found breaks in mean *Q*-values at *Q* = 0.2 and 0.8 and therefore defined individuals with mean *Q*-values from 0.2 to 0.8 as potentially admixed [22], [23], [70], [83], [89]. We found 20 individuals with mean *Q* values between 0.2 and 0.8 (Figure S2), and 10 were also assigned in GENECLASS to >1 cluster with a high probability (>0.2) of assignment to a cluster other than the origin (Table 2). In one case (individual K1, from Chirraca), an individual had low probability of being in either cluster as estimated by GENECLASS, but STRUCTURE identified it as a potential migrant (*P* = 0.046). We interpreted this individual as potentially admixed, or a migrant from an unsampled ‘ghost’ population.

We found no evidence of a bottleneck from the microsatellite data. None of the sign or Wilcoxon tests across any models suggested heterozygosity excess. Similarly, all mode-shift tests showed normal L-shaped distributions.

### Landscape Genetic Analyses

**Figure 3:**
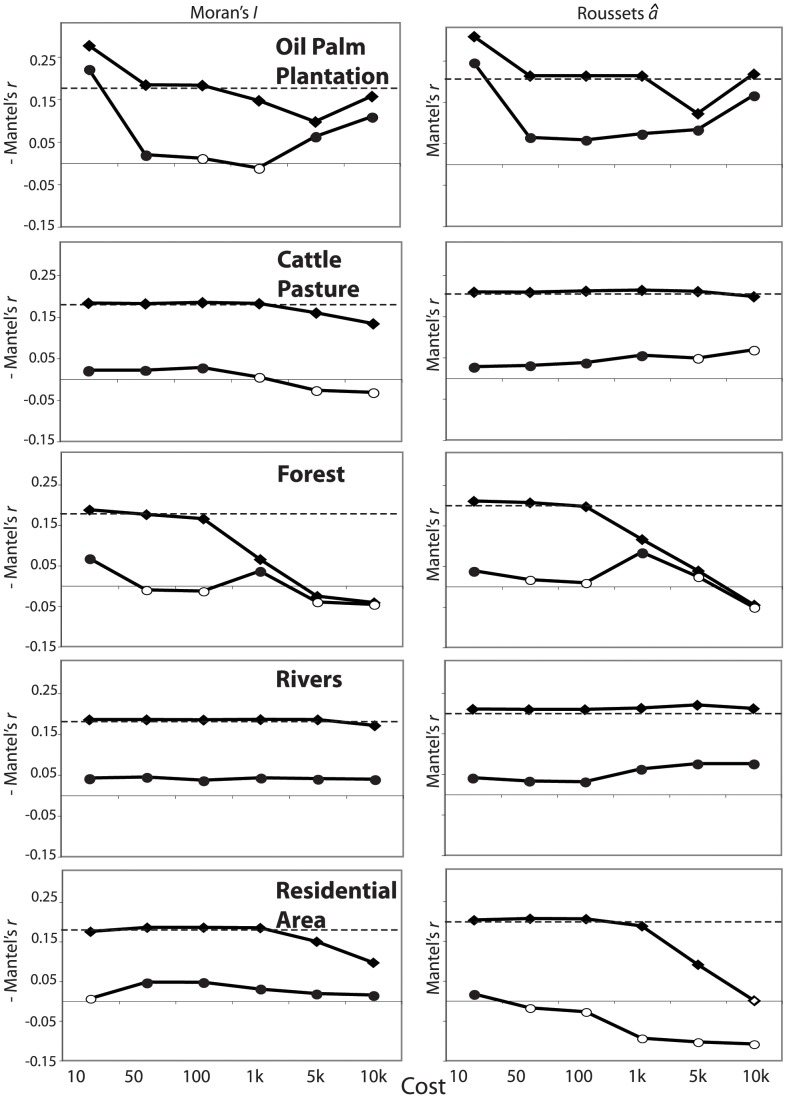
Results of Mantel tests of least-cost distances against genetic relationships, including all pairs of individuals. Results of Mantel tests of least-cost distances against genetic relationships, including all pairs of individuals. Negative Mantel’s *r-*values are given (left) for Morans’ *I* for easier comparison with trends in Rousset’s *â* (right). Filled symbols (diamonds for simple Mantel tests and circles for partial Mantel tests) represent statistically significant Mantel’s *r-*values in the expected direction (positive for *â* and negative for *I*). Dotted lines represent the Mantel’s *r-*value for Euclidean distance against genetic distance.

**Figure 4:**
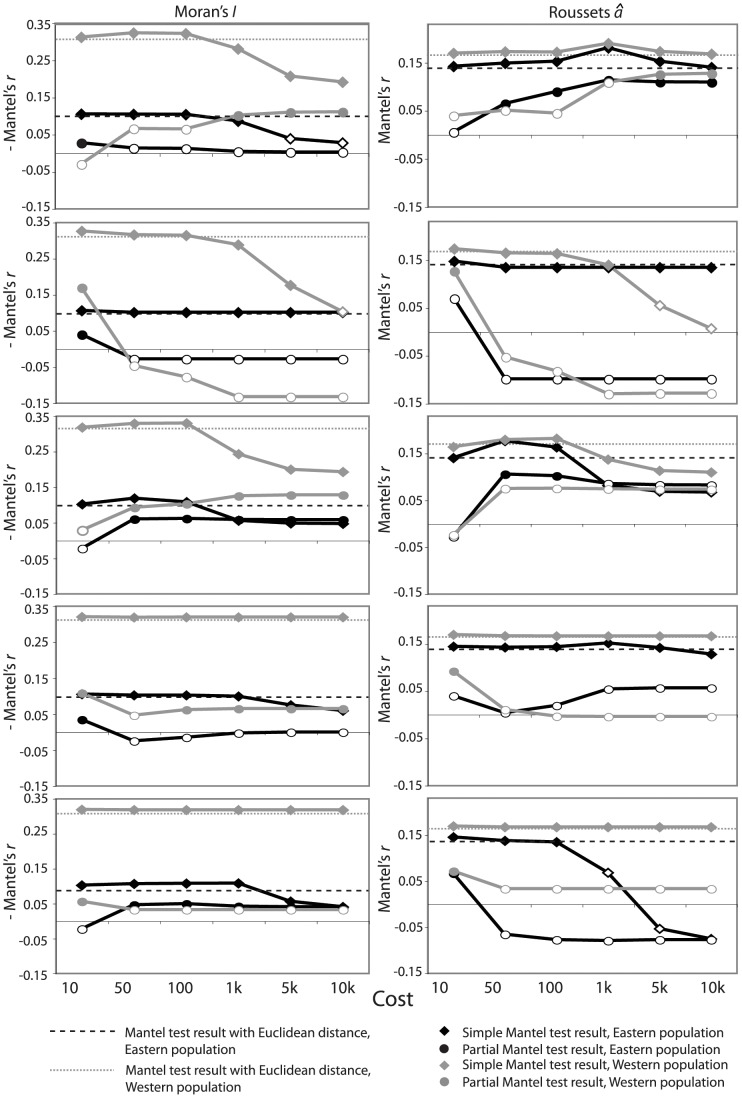
Results of Mantel tests of least-cost distances against genetic relationships, including only pairs within populations. Results of Mantel tests of least-cost distances against genetic relationships, including only pairs within the eastern population (black) or western population (grey). Least-cost distances are for oil palm plantations, cattle pastures, forests, rivers, and residential areas (top to bottom). Negative Mantel’s *r*-values are given (left) for Morans’ *I* for easier comparison with trends in Rousset’s *â* (right). Filled symbols represent statistically significant Mantel’s *r*-values in the expected direction (positive for *â* and negative for *I*).

Genetic distances were correlated with Euclidean geographic distances both within populations (eastern population, *â*: *r* = 0.14, *P* = 0.0021; *I*: *r* = −0.10, *P*<0.0001; western population, *â*: *r* = 0.17, *P*<0.0001; *I*: *r* = −0.32, *P*<0.0001) and when the entire sample was considered (*â*: *r* = 0.21, *P*<0.0001; *I*: *r* = −0.18, *P*<0.0001). Across the entire sample, oil palm plantations at a cost of 10 were the only habitat class for which Mantel’s *r*-values between least-cost and genetic distance matrices were consistently larger than Mantel’s *r*-values between genetic and Euclidean distance matrices; these results held for both Moran’s *I* and Rousset’s *â* and in both simple and partial Mantel tests (Figure 3, Table S2). However, when only within population pairs were considered, Mantel’s *r*-values between least-cost and genetic distance matrices for oil palm plantations did not differ greatly from Mantel’s *r*-values between genetic and Euclidean distance matrices (Figure 4, Table S3,S4). For both the eastern and western within population pairs, there were large, significant partial Mantel’s *r*-values for Rousset’s *â* when costs of 5,000 and 10,000 were given to oil palm plantations, but they were not greater than the Mantel’s *r*-value between genetic and Euclidean distance matrices, and Moran’s *I* did not show the same pattern (Figure 4, Table S3,S4).

Least-cost distances for forests showed the expected relationship for non-barrier habitat classes when all sample pairs were considered, with absolute Mantel’s *r*-values almost consistently decreasing with increasing cost in both simple and partial Mantel tests (Figure 3, Table S2). This pattern was less clear when only within population pairs were considered (Figure 4, Table S3,S4).

Pairwise resistance distances calculated in CIRCUITSCAPE showed strong relationships with genetic distance when all samples were considered (simple Mantel, *â*: *r* = 0.29, *P*<0.0001; *I*: *r* = −0.25, *P*<0.0001; partial Mantel controlling for Euclidean distance, *â*: *r* = 0.24, *P*<0.0001; *I*: *r* = −0.21, *P*<0.0001). Relationships were not as strong when only within population pairs were considered (eastern population, simple Mantel *â*: *r* = 0.17, *P* = 0.001; *I*: *r* = −0.11, *P*<0.0001; partial Mantel *â*: *r* = 0.09, *P*>0.05; *I*: *r* = −0.04, *P* = 0.042; western population, simple Mantel *â*: *r* = 0.14, *P* = 0.02; *I*: *r* = −0.23, *P*<0.0001; partial Mantel *â*: *r* = 0.07, *P*>0.05; *I*: *r* = −0.08, *P*>0.05). When controlling for the effect of one another, resistance distances and least-cost distances for oil palm plantations at a cost of 10 both showed significant relationships with genetic distances (Table 3). However, least-cost distances showed stronger relationships with genetic distances when controlling for resistance distances than vice versa, at least when all pairs or only western pairs were considered (Table 3).

**Table 3:** Results of partial Mantel tests between genetic distances (Moran’s *I* and Rousset’s *â*), resistance distances (generated in CIRCUITSCAPE), and the best least-cost distances (oil palm plantations at a cost of 10, “Palm10”).

	Resistance controlling for Palm 10				Palm 10 controlling for Resistance			
	Moran’s *I*		Rousset’s *â*		Moran’s *I*		Rousset’s *â*	
	Mantel’s *r*	*P*	Mantel’s *r*	*P*	Mantel’s *r*	*P*	Mantel’s *r*	*P*
All pairs	−0.1409	0.0001	0.1716	0.0002	−0.1833	0.0001	0.2004	0.0001
Eastern pairs	−0.0345	0.0524	0.0959	0.0483	−0.0287	0.0809	−0.0019	NS
Western pairs	−0.0973	0.0385	0.0726	NS	−0.2345	0.0001	0.1145	0.0099

**Figure 5:**
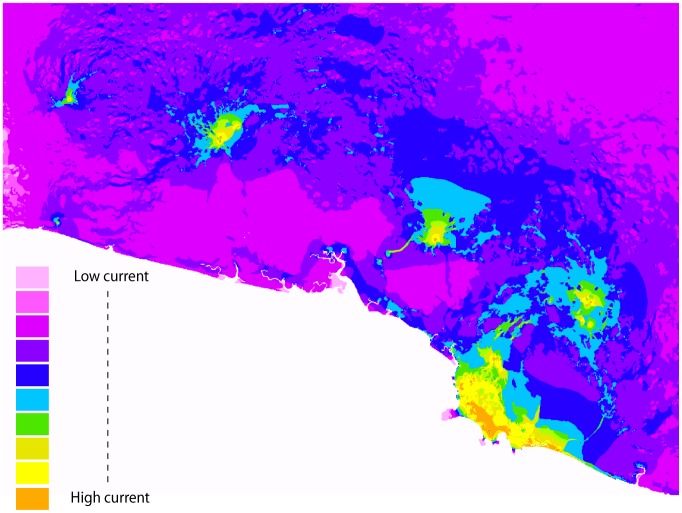
Cumulative resistance surface created in CIRCUITSCAPE. Cumulative resistance surface created in CIRCUITSCAPE. Forests, rivers, residential areas and cattle pastures were given very low resistance values (<10) and oil palm plantations were given a moderate resistance of 20.

The resistance surface output from CIRCUITSCAPE showed that even with a moderate cost, oil palm plantations cause an extensive area of low current flow in the middle of the landscape due to the cumulative costs of traversing through this expansive matrix habitat type (Figure 5). The resistance surface also shows that current flow is stronger among sites in the eastern population than in the western population (Figure 5), which is consistent with the consistently larger Mantel’s *r*-values between genetic and geographic distance matrices in the western population (Figure 4).

## Discussion

### Population Genetic Structure

Clustering analysis and F-statistics revealed that *S. o. citrinellus* are structured into two genetically distinct populations, an eastern and a western population. These populations have also been supported by AMOVAs as well as mtDNA haplogroups [42], [43]. Genetic differentiation between populations was significant but weak, which is consistent with recent separation or a high level of gene flow between them. There was a high level of agreement between clustering algorithms in terms of assignment of individuals to the two subpopulations, including the inference of recent migrants, despite the different algorithms that each program uses to describe population membership (GENECLASS calculates the probability that an individual belongs to a population, whereas STRUCTURE calculates the proportion of an individual’s genome that is characteristic of a population; Table 2). These results suggest the two populations are connected by gene flow despite their genetic distinctiveness. Importantly, individuals inferred to be migrants or of mixed or ambiguous ancestry were scattered throughout the sampled area, indicating weak differentiation across the landscape (Figure 1, Table 2).

We note that our sample contains more individuals from the eastern population, which contains more contiguous forest patches, and fewer individuals from the western population, where forest patches are less contiguous and more isolated. STRUCTURE and BAPS have been shown to produce spurious results if samples are obtained unevenly from a continuous population [94]. Also, previous publications based on simulation results have suggested that clustering analyses will group all individuals from the largest, most continuous region together, while grouping all other individuals in a second, less well resolved cluster [95]. However, this was not the case in our results; the western population, which was less continuously sampled due to the natural distribution of *S. o. citrinellus* in the area, showed a more resolved cluster than the eastern population, which showed a less well resolved cluster with evidence of ancestral polymorphism.

### Landscape Genetic Analysis

In addition to significant differentiation among populations, we also found significant genetic differentiation among several groups of *S. o. citrinellus* (but not all) using pairwise F_ST_ comparisons. These groups are in fragments of secondary forest separated by varying types of unsuitable habitats such as cattle pasture, African oil palm plantations, and rice plantations. While F-statistics effectively measure spatial *variance* in gene frequencies, we used an individual-based landscape genetic approach to instead measure aspects of spatial *patterns* of gene frequencies while taking into account landscape heterogeneity to better understand the forces behind the patterns of population genetic structure shown here.

Our results suggest that landscape heterogeneity affects genetic relationships in *S. o. citrinellus* and that different matrix habitat classes have different effects on dispersal in the studied landscape. Least-cost distances for oil palm plantations at a cost of 10 had stronger relationships with genetic distances than Euclidean distances in both simple and, more importantly, partial Mantel tests, suggesting that these least-cost distances contributed different information from Euclidean distances and that oil palm plantations represent moderate barriers to gene flow. When we restricted the analyses to within population pairs, none of the least-cost distances had stronger effects than Eucidean distances, suggesting that the effect of oil palm plantations on gene flow is largely scale-dependent and manifests during longer dispersal events between populations.

Similarly, the resistance surface showed how oil palm plantations, even when given only moderate resistance values, impede current flow because they dominate the landscape and the cost of crossing them accumulates over large distances. The strongest current flow in the resistance surface was in the eastern population near Manuel Antonio National Park (MANP), where there is not only the highest density of natural forest and monkey groups in the region, but also a break in the oil palm plantations due to complex topography. We found weaker isolation by distance in the eastern population as compared to the western population, also likely due to this break in the oil palm plantations.

By contrast, we found that cattle pastures, rivers, and residential areas do not differ greatly from Euclidean distance in their effects on genetic distance. Cattle pastures, rivers, and residential areas in this region are often surrounded by live fences of fruiting trees, which might explain why they did not show strong negative effects on *S. o. citrinellus* gene flow in this landscape. Alternatively, the landscape composition and configuration of these habitat types is quite different from that of oil palm plantations, which occur as large, contiguous expanses. Cattle pastures, rivers, and residential areas, by contrast, are smaller and more isolated from one another. In this particular landscape, these features did not influence gene flow, but if we tested multiple landscapes with a range of variability in the composition and configuration of landscape features, we might find different results [80], [86], [88].

We can lend further support to this idea by examining differences in landscape configuration between the eastern and western populations in our dataset. For example, in the western population, increasing cost for residential areas did not change Mantel’s *r*-values between least-cost and genetic distances, but in the eastern population, residential areas showed a non-barrier pattern, with increasing costs resulting in decreasing Mantel’s *r*-values. It is likely that residential areas are non-barriers in both populations, but this analysis was not sensitive enough to pick up the signal in the western population, where residential areas are smaller in number and more spread out in comparison to the eastern population.

A disadvantage to the Mantel test framework is that it is difficult to choose among closely related models or models with only slightly different Mantel’s *r*-values [96]. Here, pairwise resistance distances created in CIRCUITSCAPE produced similar Mantel’s *r*-values to least-cost distances for oil palm plantations at a cost of 10. Ours is one of a small number of empirical studies that has used CIRCUITSCAPE to characterize landscape connectivity in human-dominated environments, and the other studies have found that resistance distances outperform least-cost distances in how well they characterize gene flow [92], [97]. Here, partial Mantel tests controlling for the effect of least-cost distances on resistance and vice versa suggested that oil palm plantations at a cost of 10 had the stronger relationship with genetic distance when all pairs were considered and when only western pairs were considered (Table 3). However, the Mantel’s *r*-values were within a reasonable margin of error of one another. Both distances assigned moderate costs to oil palm plantations and had stronger relationships with genetic distances than Euclidean distance matrices when between population pairs were included in the analysis, showing a consistent trend.

It is possible that the temporal scale of our landscape data has influenced our results [98], [99]. Long, overlapping generations affect the power of landscape genetic approaches to detect the effects of current or even historical landscape patterns on genetic structure, and this issue is particularly important for long-lived primate taxa. A recent simulation study showed that it would take 1–15 generations to detect barriers to gene flow using Mantel’s *r* [99]. *Saimiri* have an average generation time of 3–6 years [100], meaning that at least 12 and up to 25 generations have likely transpired since the major transformation of the Central Pacific landscape in the early 1900s (although at that time the plantations were of banana, and later replaced with African oil palm, they were of the same configuration and area) [38]. Thus, we should have been able to detect barriers to gene flow caused by landscape features in this study. However, genetic distance measures such as Rousset’s *â* are based on F_ST_ and as such may reflect processes that are more likely to be apparent in historical landscape data [4]. Although we have shown some effect of landscape heterogeneity on gene flow using current landscape data, historical data from the early 1900s may show a stronger relationship. Unfortunately, such data not readily available.

Although we tested for a population contraction and found no evidence for a bottleneck, sequencing additional loci, in particular nuclear introns and coding mtDNA loci, would allow for a more robust analysis of alternative hypotheses through Approximate Bayesian Computation (ABC) [101]. ABC would allow us to test various alternative scenarios of population expansion and/or contraction and also to test for a temporal correlation between increasing genetic structure and the expansion of banana and oil palm plantations in the Central Pacific of Costa Rica.

Another issue that is common in many landscape genetic studies is that the different focal matrix habitats used in our analysis are likely of different ages. Rivers are older than the other four habitat classes we considered, and some residential areas are likely younger than the cattle pastures and oil palm plantations, most of which were established in the early 1900s. Future analyses might address this issue by including different sets of models and molecular markers that pinpoint different temporal scales in order to distinguish between the effects of historical and recent landscape changes on population genetic structure [102].

### Implications for Conservation Management

This study exemplifies how important it is to conduct landscape genetic analyses in species that show evidence of population genetic structure in heterogeneous landscapes. Furthermore, we highlight the importance of quantifying the relative effects of different matrix habitat classes in landscape genetic analyses [4], [18], [103], instead of assuming that all non-suitable habitats have a uniform effect on dispersal and gene flow. Because we distinguished among matrix habitat classes, we have a finer understanding of what does and does not constitute a barrier to *S. o. citrinellus* gene flow in the Central Pacific Costa Rican landscape. We are also able to make more detailed recommendations to conservation managers regarding the types of matrix habitat that *S. o. citrinellus* may or may not use to disperse among patches of forest in the Central Pacific. In a concurrent study, we used resistance surfaces to test different biological corridor configurations for their potential ability to augment gene flow through oil palm plantations [42]. Another strategy to augment gene flow through oil palm plantations might be to plant understory vegetation, which has been shown to increase bird richness in oil palm plantations in eastern Guatemala [104].

However, we must recognize that our results are specific to the Central Pacific landscape, and conservation managers should be careful not to apply our results in other landscapes or for other populations of *S. oerstedii*. For example, in a landscape where cattle pastures dominate instead of oil palm plantations, a separate landscape genetic study would be necessary to measure the relative effects of each matrix habitat to determine whether it might be more important for conservation managers to augment gene flow through cattle pastures rather than oil palm plantations.

When attempting to translate the results of any landscape genetic analysis to patterns of functional connectivity, we must also acknowledge that measures of genetic distance do not equate to animal movement patterns. Simulations that model the sums of individual behavioral decisions are likely necessary to best inform conservation management of taxa in heterogeneous landscapes [105], [106], [107], [108]. A possible next step would be to incorporate a recently published new form of population viability analysis that uses individual-based models (simulations) that incorporate behavioral decisions alongside models of landscape change over time [109].

## Supporting Information

Figure S1 **Inference of the number of genetic clusters (K) estimated using STRUCTURE.**
(DOC)

Figure S2 **Ranked mean** ***Q*** **(proportional membership in each cluster) for each individual.**
(DOC)

Table S1 **Microsatellite markers amplified in 244** ***Saimiri oerstedii*** **samples.**
(DOC)

Table S2 **Results of simple and partial Mantel tests between genetic distances (Moran’s** ***I*** **and Rousset’s** ***â*** **) and cost distances among all individuals.**
(DOC)

Table S3 **Results of simple and partial Mantel tests between genetic distances (Moran’s** ***I*** **and Rousset’s** ***â*** **) and cost distances, including only sample pairs within the eastern population.**
(DOC)

Table S4 **Results of simple and partial Mantel tests between genetic distances (Moran’s** ***I*** **and Rousset’s** ***â*** **) and cost distances, including only sample pairs within the western population.**
(DOC)
