# Supplementary material for: Scale-Dependent Effects of a Heterogeneous Landscape on Genetic Differentiation in the Central American Squirrel Monkey (Saimiri oerstedii)
Source: PLoS One. 2012 Aug 15;7(8):e43027. doi: 10.1371/journal.pone.0043027 (PMC3419685; doi:10.1371/journal.pone.0043027)
Supplement: Figure S1 — Inference of the number of genetic clusters (K) estimated using STRUCTURE. (DOC) [file pone.0043027.s001.doc]

**Figure S1.** Inference of the number of genetic clusters (K) estimated using STRUCTURE. Both ln *P*(X | K) (the likelihood of the data given K; small diamonds) and ΔK (the standardized second order rate of change of ln *P*(X | K); black circles) are plotted as a function of K. Error bars of ln *P*(X | K) represent standard deviations.
