# Supplementary material for: Scale-Dependent Effects of a Heterogeneous Landscape on Genetic Differentiation in the Central American Squirrel Monkey (Saimiri oerstedii)
Source: PLoS One. 2012 Aug 15;7(8):e43027. doi: 10.1371/journal.pone.0043027 (PMC3419685; doi:10.1371/journal.pone.0043027)
Supplement: Figure S2 — Ranked mean Q (proportional membership in each cluster) for each individual. (DOC) [file pone.0043027.s002.doc]

**Figure S2.** Ranked mean *Q* (proportional membership in each cluster) for each individual in each *S. o. citrinellus* cluster (Western – black triangles, or Eastern – white squares) estimated in STRUCTURE. Admixed individuals have mean *Q* values between 0.2 and 0.8.
