# Supplementary material for: Scale-Dependent Effects of a Heterogeneous Landscape on Genetic Differentiation in the Central American Squirrel Monkey (Saimiri oerstedii)
Source: PLoS One. 2012 Aug 15;7(8):e43027. doi: 10.1371/journal.pone.0043027 (PMC3419685; doi:10.1371/journal.pone.0043027)
Supplement: Table S1 — Microsatellite markers amplified in 244 Saimiri oerstedii samples. (DOC) [file pone.0043027.s003.doc]

**Table S1. Microsatellite markers amplified in 244 *Saimiri oerstedii* samples.**

| **Marker** | **No. of Alleles** | **Allele Size Range** | **H_o_^*^** | **Repeat Type** | **Reference** |
| --- | --- | --- | --- | --- | --- |
| CJ7 | 11 | 130-150 | 0.668 | di | [55] |
| D17s804 | 18 | 132-202 | 0.414 | di | [56] |
| D3s1210 | 7 | 117-131 | 0.119 | di | [56] |
| D3s1229 | 18 | 84-132 | 0.652 | di | [56] |
| D3s1766 | 8 | 187-226 | 0.557 | tetra | [56] |
| D4s111 | 18 | 130-168 | 0.398 | di | [56] |
| D5s111 | 7 | 155-179 | 0.402 | di | [56] |
| D8s165 | 13 | 137-163 | 0.467 | di | [56] |
| D8s260 | 10 | 219-241 | 0.676 | di | [56] |
| Leon15 | 7 | 262-280 | 0.488 | di | [57] |
| Leon21 | 14 | 326-386 | 0.480 | di | [57] |
| LL118 | 14 | 110-158 | 0.373 | di | [58] |
| LL157 | 10 | 207-239 | 0.443 | di | [58] |
| LL311 | 31 | 212-317 | 0.730 | tri | [58] |
| Locus5 | 9 | 102-118 | 0.316 | di | [59] |
| SB38 | 6 | 133-145 | 0.475 | di | [60] |

^*^Observed heterozygosity
