# Supplementary material for: Scale-Dependent Effects of a Heterogeneous Landscape on Genetic Differentiation in the Central American Squirrel Monkey (Saimiri oerstedii)
Source: PLoS One. 2012 Aug 15;7(8):e43027. doi: 10.1371/journal.pone.0043027 (PMC3419685; doi:10.1371/journal.pone.0043027)
Supplement: Table S2 — Results of simple and partial Mantel tests between genetic distances (Moran’s I and Rousset’s â ) and cost distances among all individuals. (DOC) [file pone.0043027.s004.doc]

**Table S2**. Results of simple and partial Mantel tests between genetic distances (Moran’s *I* and Rousset’s *â*) and cost distances among all individuals.

|  | **Mantel Tests** | |  |  | **Partial Mantel Tests** | | |  |
| --- | --- | --- | --- | --- | --- | --- | --- | --- |
|  | **Moran's *I*** | | **Rousset's *â*** | | **Moran's *I*** | | **Rousset's *â*** | |
| **Cost-Distance** | **Mantel's *r*** | ***P*** | **Mantel's *r*** | ***P*** | **Mantel's *r*** | ***P*** | **Mantel's *r*** | ***P*** |
| Palm10* | -0.2759 | 0.0001 | 0.3074 | 0.0001 | -0.2201 | 0.0001 | 0.2442 | 0.0001 |
| Palm50 | -0.1830 | 0.0001 | 0.2125 | 0.0001 | -0.0186 | 0.0024 | 0.0638 | 0.0229 |
| Palm100 | -0.1819 | 0.0001 | 0.2127 | 0.0001 | -0.0102 | NS | 0.0578 | 0.0423 |
| Palm1k | -0.1465 | 0.0001 | 0.2126 | 0.0001 | 0.0125 | 0.0397 | 0.0724 | 0.0353 |
| Palm5k | -0.0963 | 0.0001 | 0.1215 | 0.0001 | -0.0613 | 0.0001 | 0.0825 | 0.0005 |
| Palm10k | -0.1561 | 0.0001 | 0.2169 | 0.0001 | -0.1078 | 0.0001 | 0.1654 | 0.0001 |
| Cattle10 | -0.1824 | 0.0001 | 0.2082 | 0.0001 | -0.0207 | 0.0004 | 0.0266 | 0.0155 |
| Cattle50 | -0.1809 | 0.0001 | 0.2075 | 0.0001 | -0.0206 | 0.0004 | 0.0298 | 0.0081 |
| Cattle100 | -0.1838 | 0.0001 | 0.2103 | 0.0001 | -0.0270 | 0.0001 | 0.0367 | 0.0036 |
| Cattle1k | -0.1812 | 0.0001 | 0.2123 | 0.0001 | -0.0043 | NS | 0.0546 | 0.0324 |
| Cattle5k | -0.1586 | 0.0001 | 0.2097 | 0.0001 | 0.0278 | 0.0001 | 0.0479 | NS |
| Cattle10k | -0.1327 | 0.0001 | 0.1970 | 0.0001 | 0.0330 | 0.0001 | 0.0677 | NS |
| Forest10 | -0.1871 | 0.0001 | 0.2097 | 0.0001 | -0.0670 | 0.0001 | 0.0378 | 0.0408 |
| Forest50 | -0.1755 | 0.0001 | 0.2058 | 0.0001 | 0.0111 | NS | 0.0161 | NS |
| Forest100 | -0.1651 | 0.0001 | 0.1962 | 0.0001 | 0.0138 | 0.0186 | 0.0080 | NS |
| Forest1k | -0.0649 | 0.0001 | 0.1154 | 0.0001 | -0.0358 | 0.0001 | 0.0839 | 0.0019 |
| Forest5k | 0.0262 | 0.0001 | 0.0380 | 0.0001 | 0.0405 | 0.0001 | 0.0231 | NS |
| Forest10k | 0.0419 | 0.0001 | -0.0462 | 0.0001 | 0.0471 | 0.0001 | -0.0522 | 0.0100 |
| Rivers10 | -0.1844 | 0.0001 | 0.2094 | 0.0001 | -0.0415 | 0.0001 | 0.0404 | 0.0138 |
| Rivers50 | -0.1844 | 0.0001 | 0.2088 | 0.0001 | -0.0441 | 0.0001 | 0.0322 | 0.0209 |
| Rivers100 | -0.1840 | 0.0001 | 0.2087 | 0.0001 | -0.0360 | 0.0001 | 0.0303 | 0.0395 |
| Rivers1k | -0.1852 | 0.0001 | 0.2122 | 0.0001 | -0.0421 | 0.0001 | 0.0617 | 0.0115 |
| Rivers5k | -0.1846 | 0.0001 | 0.2198 | 0.0001 | -0.0402 | 0.0001 | 0.0750 | 0.0086 |
| Rivers10k | -0.1702 | 0.0001 | 0.2112 | 0.0001 | -0.0388 | 0.0001 | 0.0751 | 0.0085 |
| Residential10 | -0.1747 | 0.0001 | 0.2019 | 0.0001 | -0.0056 | NS | 0.0164 | 0.0863 |
| Residential50 | -0.1851 | 0.0001 | 0.2052 | 0.0001 | -0.0471 | 0.0001 | -0.0185 | NS |
| Residential100 | -0.1851 | 0.0001 | 0.2044 | 0.0001 | -0.0466 | 0.0001 | -0.0284 | NS |
| Residential1k | -0.1843 | 0.0001 | 0.1869 | 0.0001 | -0.0295 | 0.0001 | -0.0946 | 0.0058 |
| Residential5k | -0.1495 | 0.0001 | 0.0903 | 0.0001 | -0.0181 | 0.0022 | -0.1040 | 0.0040 |
| Residential10k | -0.0963 | 0.0001 | 0.0005 | NS | -0.0144 | 0.0134 | -0.1091 | 0.0024 |

*Palm10 = Oil Palm plantations given a cost of 10, 5k represents a cost of 5,000

NS= Not significant
