# Supplementary material for: Scale-Dependent Effects of a Heterogeneous Landscape on Genetic Differentiation in the Central American Squirrel Monkey (Saimiri oerstedii)
Source: PLoS One. 2012 Aug 15;7(8):e43027. doi: 10.1371/journal.pone.0043027 (PMC3419685; doi:10.1371/journal.pone.0043027)
Supplement: Table S3 — Results of simple and partial Mantel tests between genetic distances (Moran’s I and Rousset’s â ) and cost distances, including only sample pairs within the eastern population. (DOC) [file pone.0043027.s005.doc]

**Table S3**. Results of simple and partial Mantel tests between genetic distances (Moran’s *I* and Rousset’s *â*) and cost distances, including only sample pairs within the eastern population.

|  | **Mantel Tests** | |  |  | **Partial Mantel Tests** | | |  |
| --- | --- | --- | --- | --- | --- | --- | --- | --- |
|  | **Moran's *I*** | | **Rousset's *a*** | | **Moran's *I*** | | **Rousset's *a*** | |
| **Cost-Distance** | **Mantel's *r*** | ***P*** | **Mantel's *r*** | ***P*** | **Mantel's *r*** | ***P*** | **Mantel's *r*** | ***P*** |
| Palm10* | -0.1049 | 0.0001 | 0.1420 | 0.0018 | -0.0275 | 0.0356 | 0.0052 | NS |
| Palm50 | -0.1040 | 0.0001 | 0.1484 | 0.0015 | -0.0128 | NS | 0.0643 | 0.0230 |
| Palm100 | -0.1039 | 0.0001 | 0.1523 | 0.0011 | -0.0119 | NS | 0.0897 | 0.0251 |
| Palm1k | -0.0862 | 0.0002 | 0.1808 | 0.0019 | -0.0037 | NS | 0.1133 | 0.0438 |
| Palm5k | -0.0390 | NS | 0.1522 | 0.0219 | -0.0022 | NS | 0.1098 | 0.0482 |
| Palm10k | -0.0275 | NS | 0.1399 | 0.0256 | -0.0020 | NS | 0.1092 | 0.0543 |
| Cattle10 | -0.1057 | 0.0001 | 0.1465 | 0.0012 | -0.0393 | 0.0184 | 0.0687 | NS |
| Cattle50 | -0.1006 | 0.0001 | 0.1337 | 0.0050 | 0.0283 | NS | -0.0992 | 0.0148 |
| Cattle100 | -0.1006 | 0.0001 | 0.1337 | 0.0045 | 0.0283 | NS | -0.0992 | 0.0155 |
| Cattle1k | -0.1006 | 0.0001 | 0.1337 | 0.0043 | 0.0283 | NS | -0.0992 | 0.0152 |
| Cattle5k | -0.1006 | 0.0001 | 0.1337 | 0.0050 | 0.0283 | NS | -0.0992 | 0.0163 |
| Cattle10k | -0.1006 | 0.0001 | 0.1337 | 0.0044 | 0.0283 | NS | -0.0992 | 0.0159 |
| Forest10 | -0.1016 | 0.0001 | 0.1400 | 0.0023 | 0.0227 | 0.0514 | -0.0279 | NS |
| Forest50 | -0.1183 | 0.0010 | 0.1763 | 0.0001 | -0.0598 | 0.0016 | 0.1056 | 0.0164 |
| Forest100 | -0.1082 | 0.0001 | 0.1631 | 0.0001 | -0.0613 | 0.0017 | 0.1021 | 0.0248 |
| Forest1k | -0.0559 | 0.0061 | 0.0816 | NS | -0.0585 | 0.0057 | 0.0856 | NS |
| Forest5k | -0.0478 | 0.0165 | 0.0686 | NS | -0.0578 | 0.0046 | 0.0828 | NS |
| Forest10k | -0.0468 | 0.0159 | 0.0669 | NS | -0.0578 | 0.0040 | 0.0825 | NS |
| Rivers10 | -0.1048 | 0.0001 | 0.1438 | 0.0012 | -0.0342 | 0.0202 | 0.0393 | NS |
| Rivers50 | -0.1019 | 0.0001 | 0.1420 | 0.0025 | 0.0251 | NS | 0.0037 | NS |
| Rivers100 | -0.1019 | 0.0001 | 0.1430 | 0.0020 | 0.0152 | NS | 0.0189 | NS |
| Rivers1k | -0.0988 | 0.0001 | 0.1510 | 0.0018 | 0.0035 | NS | 0.0538 | NS |
| Rivers5k | -0.0743 | 0.0009 | 0.1412 | 0.0128 | 0.0010 | NS | 0.0559 | NS |
| Rivers10k | -0.0591 | 0.0067 | 0.1274 | 0.0271 | 0.0007 | NS | 0.0561 | NS |
| Residential10 | -0.1020 | 0.0001 | 0.1450 | 0.0031 | 0.0233 | NS | 0.0666 | 0.0523 |
| Residential50 | -0.1062 | 0.0001 | 0.1371 | 0.0029 | -0.0462 | 0.0044 | -0.0655 | NS |
| Residential100 | -0.1073 | 0.0001 | 0.1343 | 0.0028 | -0.0489 | 0.0080 | -0.0772 | NS |
| Residential1k | -0.1079 | 0.0001 | 0.0681 | NS | -0.0419 | 0.0321 | -0.0792 | NS |
| Residential5k | -0.0556 | 0.0074 | -0.0533 | NS | -0.0400 | 0.0397 | -0.0776 | NS |
| Residential10k | -0.0404 | 0.0384 | -0.0754 | NS | -0.0397 | 0.0425 | -0.0773 | NS |

*Palm10 = Oil Palm plantations given a cost of 10, 5k represents a cost of 5,000

NS= Not significant
