# Supplementary material for: Scale-Dependent Effects of a Heterogeneous Landscape on Genetic Differentiation in the Central American Squirrel Monkey (Saimiri oerstedii)
Source: PLoS One. 2012 Aug 15;7(8):e43027. doi: 10.1371/journal.pone.0043027 (PMC3419685; doi:10.1371/journal.pone.0043027)
Supplement: Table S4 — Results of simple and partial Mantel tests between genetic distances (Moran’s I and Rousset’s â ) and cost distances, including only sample pairs within the western population. (DOC) [file pone.0043027.s006.doc]

**Table S4**. Results of simple and partial Mantel tests between genetic distances (Moran’s *I* and Rousset’s *â*) and cost distances, including only sample pairs within the western population.

|  | **Mantel Tests** | |  |  | **Partial Mantel Tests** | | |  |
| --- | --- | --- | --- | --- | --- | --- | --- | --- |
|  | **Moran's *I*** | | **Rousset's *a*** | | **Moran's *I*** | | **Rousset's *a*** | |
| **Cost-Distance** | **Mantel's *r*** | ***P*** | **Mantel's *r*** | ***P*** | **Mantel's *r*** | ***P*** | **Mantel's *r*** | ***P*** |
| Palm10* | -0.3118 | 0.0001 | 0.1691 | 0.0001 | 0.0304 | NS | 0.0395 | NS |
| Palm50 | -0.3229 | 0.0001 | 0.1725 | 0.0001 | -0.0652 | NS | 0.0507 | NS |
| Palm100 | -0.3213 | 0.0001 | 0.1715 | 0.0002 | -0.0646 | NS | 0.0447 | NS |
| Palm1k | -0.2803 | 0.0001 | 0.1895 | 0.0021 | -0.1015 | 0.0331 | 0.1086 | NS |
| Palm5k | -0.2067 | 0.0001 | 0.1729 | 0.0130 | -0.1094 | 0.0256 | 0.1250 | 0.0573 |
| Palm10k | -0.1908 | 0.0007 | 0.1675 | 0.0154 | -0.1104 | 0.0266 | 0.1272 | 0.0574 |
| Cattle10 | -0.3255 | 0.0001 | 0.1726 | 0.0001 | -0.1690 | 0.0001 | 0.1259 | 0.0267 |
| Cattle50 | -0.3154 | 0.0001 | 0.1640 | 0.0001 | 0.0466 | 0.0384 | -0.0532 | NS |
| Cattle100 | -0.3141 | 0.0001 | 0.1627 | 0.0001 | 0.0779 | 0.0054 | -0.0826 | 0.0309 |
| Cattle1k | -0.2878 | 0.0001 | 0.1390 | 0.0005 | 0.1331 | 0.0005 | -0.1302 | 0.0310 |
| Cattle5k | -0.1758 | 0.0003 | 0.0549 | NS | 0.1331 | 0.0008 | -0.1290 | 0.0352 |
| Cattle10k | -0.1027 | NS | 0.0065 | NS | 0.1329 | 0.0007 | -0.1287 | 0.0354 |
| Forest10 | -0.3180 | 0.0001 | 0.1640 | 0.0001 | -0.0284 | NS | -0.0238 | NS |
| Forest50 | -0.3289 | 0.0001 | 0.1794 | 0.0001 | -0.0927 | 0.0245 | 0.0750 | NS |
| Forest100 | -0.3297 | 0.0001 | 0.1816 | 0.0001 | -0.1031 | 0.0144 | 0.0752 | NS |
| Forest1k | -0.2432 | 0.0001 | 0.1371 | 0.0124 | -0.1249 | 0.0054 | 0.0739 | NS |
| Forest5k | -0.1994 | 0.0001 | 0.1132 | 0.0427 | -0.1275 | 0.0055 | 0.0735 | NS |
| Forest10k | -0.1929 | 0.0001 | 0.1096 | 0.0519 | -0.1279 | 0.0060 | 0.0734 | NS |
| Rivers10 | -0.3203 | 0.0001 | 0.1688 | 0.0001 | -0.1079 | 0.0010 | 0.0918 | 0.0480 |
| Rivers50 | -0.3183 | 0.0001 | 0.1661 | 0.0001 | -0.0463 | NS | 0.0104 | NS |
| Rivers100 | -0.3189 | 0.0001 | 0.1656 | 0.0001 | -0.0617 | 0.0302 | -0.0035 | NS |
| Rivers1k | -0.3190 | 0.0001 | 0.1655 | 0.0001 | -0.0644 | 0.0284 | -0.0045 | NS |
| Rivers5k | -0.3190 | 0.0001 | 0.1655 | 0.0001 | -0.0644 | 0.0274 | -0.0045 | NS |
| Rivers10k | -0.3190 | 0.0001 | 0.1655 | 0.0001 | -0.0644 | 0.0267 | -0.0045 | NS |
| Residential10 | -0.3190 | 0.0001 | 0.1688 | 0.0001 | -0.0557 | 0.0191 | 0.0715 | 0.0182 |
| Residential50 | -0.3179 | 0.0001 | 0.1670 | 0.0001 | -0.0320 | NS | 0.0329 | NS |
| Residential100 | -0.3179 | 0.0001 | 0.1670 | 0.0001 | -0.0320 | NS | 0.0329 | NS |
| Residential1k | -0.3179 | 0.0001 | 0.1670 | 0.0001 | -0.0320 | NS | 0.0329 | NS |
| Residential5k | -0.3179 | 0.0001 | 0.1670 | 0.0001 | -0.0320 | NS | 0.0329 | NS |
| Residential10k | -0.3179 | 0.0001 | 0.1670 | 0.0001 | -0.0320 | NS | 0.0329 | NS |

*Palm10 = Oil Palm plantations given a cost of 10, 5k represents a cost of 5,000

NS= Not significant
